# Cardiovascular impairment in young adults horizontally infected with HIV-1 in childhood

**DOI:** 10.1186/1471-2334-13-S1-O28

**Published:** 2013-12-16

**Authors:** Florentina Dumitrescu, Dina Maria Cupşa, Andreea Cristina Stoian, Augustin Cupşa, Irina Niculescu, Lucian Giubelan, Daniela Culman

**Affiliations:** 1 University of Medicine and Pharmacy Craiova, Romania; 2 “Victor Babeş” Clinical Hospital of Infectious Diseases and Pneumology, Craiova, Romania

## Background

We aimed to evaluate the prevalence and spectrum of cardiovascular impairment (CVI) in young HIV/AIDS patients in evidence at the HIV/AIDS Craiova Regional Center.

## Methods

We performed a retrospective study between 01 January 1996 – 31 December 2005 and a prospective study between 01 June 2005 – 30 June 2010; it targeted epidemiological issues, the clinical spectrum of CVI in patient with horizontal HIV infection, acquired during 1987-1990 (lot P) compared to patients with other transmission routes of infection (lot S).

## Results

CVI was encountered in 61 (10.23%) of 596 patients parenterally-infected with HIV-1 in childhood and 32 (16.16%) of 198 patients infected through sexual route, with statistically significant differences in the prevalence of CVI depending on the transmission route of HIV-1 (pChi²=0.02), probably being underestimated in pre-HAART era. The average age at the moment of CVI was 9.02±4.76 years in lot P vs. 41.47±12.41 years in lot S (p< 0.0001). The spectrum of CVI was dominated by arrhythmias and conduction blocks 42 patients (45.16%), dilated cardiomyopathy (DCM) 18 patients (19.35%), pulmonary arterial hypertension 17 patients (18.27%). The most common entity identified in parenterally-infected patients in early childhood was DCM 16 patients lot P vs. 2 patients lot S (p Fischer test=0.026), found in moderate and severe immunosuppressed patients, with virological failure, often associated with respiratory disease. In sexually-infected patients diseases due to atherosclerosis prevailed 17 patients (p Fisher test<0.0001). The presence of high blood pressure in 2 young people with HIV/AIDS raises the issue of cardiovascular risk and occurrence of coronary heart disease in young patients. Although the survival rate of patients with CVI (15.21 ± 0.62 years) was similar to that of the patients without CVI (13.78 ± 0.25 years), for patients with CVI, death due to direct cardiac damage was significant: 9 patients (52.94%).

## Conclusion

The prevalence of CVI was low, probably overlooked and underdiagnosed, at least in early ages and young adults, dominated by arrhythmias and DCM; monitoring and management in individual care programs constitutes a way for improving the quality of life and prognosis for people with HIV/AIDS.

